# Supplementary figures and images for: Validation of reference genes for RT-qPCR relative expression analysis during cyst-to-early adult development of Taenia solium
Source: PLoS Negl Trop Dis. 2026 Jan 2;20(1):e0013893. doi: 10.1371/journal.pntd.0013893 (PMC12900441; doi:10.1371/journal.pntd.0013893)

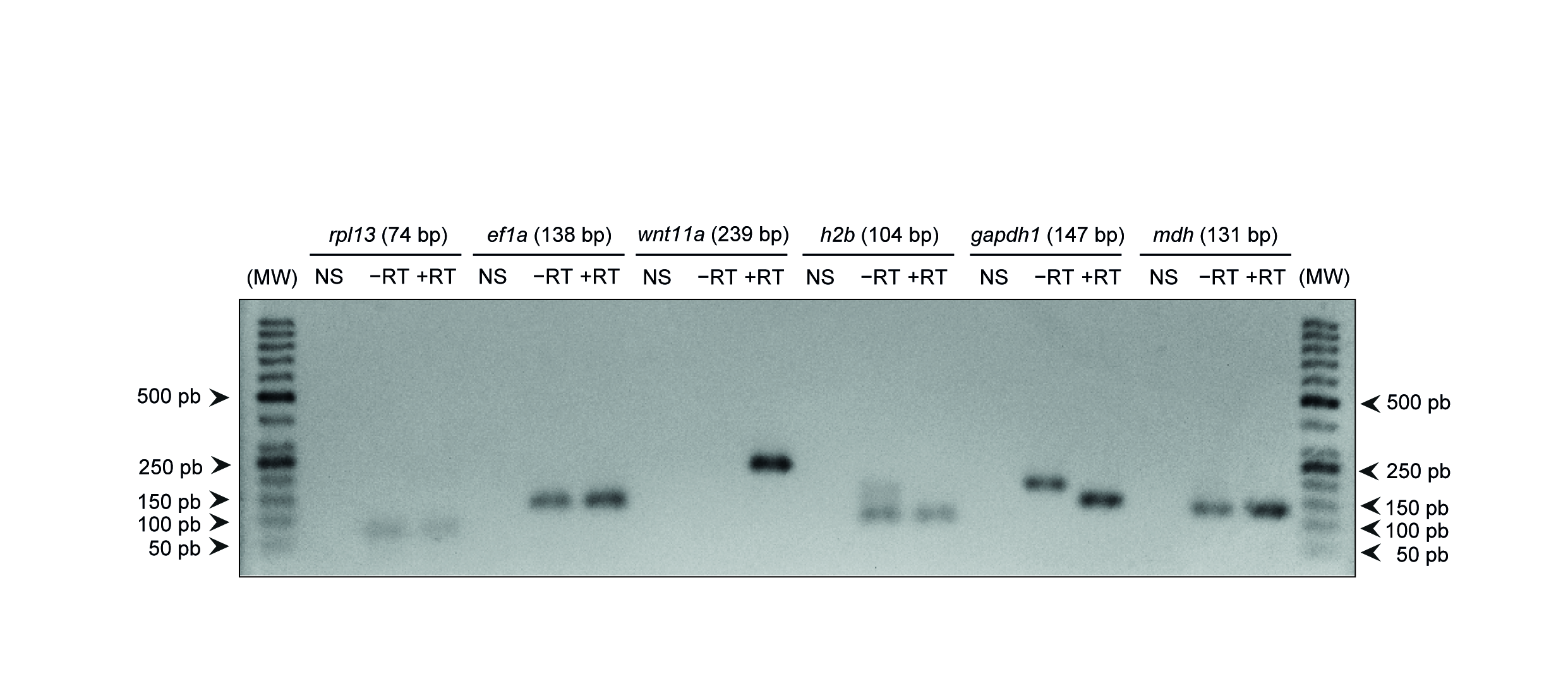

Supplement: S1 Fig — Agarose gel electrophoresis of end-point RT–PCR products for rpl13 (74 bp), ef1a (138 bp), wnt11a (239 bp), h2b (104 bp), gapdh1 (147 bp), and mdh (131 bp). For each target, three reaction conditions are shown: no-sample control (NS), reaction performed without reverse transcriptase (−RT), and reaction performed with reverse transcriptase (+RT). Molecular weight markers (MW) are shown on both sides of the gel, with fragment sizes indicated in base pairs (bp). (TIF) [file pntd.0013893.s002.tif]
