# Supplementary material for: Validation of reference genes for RT-qPCR relative expression analysis during cyst-to-early adult development of Taenia solium
Source: PLoS Negl Trop Dis. 2026 Jan 2;20(1):e0013893. doi: 10.1371/journal.pntd.0013893 (PMC12900441; doi:10.1371/journal.pntd.0013893)
Supplement: Attachment — Submitted filename: Response to Reviewers.docx [file pntd.0013893.s007.docx]

**PNTD-D-25-01312
Validation of reference genes for RT-qPCR relative expression analysis during cyst-to-early adult**

Dear editors,

We are sincerely grateful to the reviewers for their careful evaluation of our manuscript and for their insightful, encouraging comments and suggestions. A special note of thanks goes to them for their generosity in providing both positive feedback and thoughtful guidance on how to address their constructive criticism on some points. This has helped us refine the work and strengthen its rigor, thereby enhancing its usefulness for readers.

We have carefully considered all comments and revised the manuscript accordingly. Please find a point-by-point response below. We copied each reviewer´s comments in full, indenting them as paragraphs; specific requests are italicized and sometimes also underlined.

We trust that the revised manuscript addresses all concerns and enhances the contribution of our work to the field, deeming it publishable for PLoS NTD.

…………………...

**Reviewer #1**

**Methods (R#1)**

This study uses rigorous and comprehensive methods to determine suitable reference genes (RGs) for reverse transcriptase quantitative PCR. The authors use appropriate number of samples as well as technical and biological replicates. Statistical analysis was clearly stated and described in the methods, legends, and text. I highly commend the authors for their rigor.

We sincerely appreciate the reviewer’s positive assessment of our methodological approach and our commitment to analytical rigor and reproducibility.

**Results (R#1)**

The logical flow for accessing primer stabilities and unvarying expression across many conditions is very well laid out. The authors convincingly show that some common transcripts used of qPCR normalization are less than ideal. Eight primer pairs emerged as potential RGs. To experimentally validate putative RGs, the authors collected 3 stages of T. solium development in the presence or absence of an excystment trigger, taurocholic acid (TA), for which matched RNA-seq data is available. While there were variations in the trends, this is to be expected for qPCR data from complex tissues and timepoints. There was general concordance between qPCR and RNA-seq trends, especially with the preferred RGs. It is unfortunate that there aren’t clearer conditions that the authors can use to biologically validate their RGs. The difference in h2b expression +/- TA is not strikingly different even from the RNAseq data. Expression of wnt11a is even more variable but that could represent real biological variation.

Thank you for this thoughtful and encouraging assessment of our results. We appreciate the recognition of the analytical flow and the general concordance between qPCR and RNA-seq trends. We agree that the validation condition must be clear, and we assert that the TA+ condition provides the essential biological validation necessary to confirm the robustness of *rpl13* and *ef1α* as RGs.

Adding TA serves as a proxy for the natural condition within the human gut; we currently include it in further *in vitro* studies, to increase their biological relevance. TA induces evagination of the scolex and stimulates proliferation, driving the transition into the adult phase and the corresponding bodyplan. Furthermore, *mdh* (malate dehydrogenase), characteristic of adult cestodes (Ref. 48 DOI: [10.1016/j.ygeno.2021.01.005](https://doi.org/10.1016/j.ygeno.2021.01.005)), exhibited high stability only in TA+ samples. Under these conditions, normalization using *rpl13* and *ef1α* produced RT-qPCR results that aligned well with RNA-seq trends for both *h2b* and *wnt11a*, supporting the biological validity of these RGs in a synchronized context.

As elaborated in a response to Reviewer #2 about the Results (page 5) and added explicitly in the revised Discussion, we hypothesize a synchronizing role for TA. The critical finding is that normalization of *h2b* expression using the geometric mean of *rpl13* and *ef1α* aligned with RNA-seq data only under TA+ conditions.

We agree that the observed variability may reflect genuine biological dynamics within complex developmental stages, which are not sufficiently explored in *T. solium*. We selected *h2b* and *wnt11a* specifically because they are expected to be transcriptionally dynamic during proliferation and strobilation.

While the overall difference in *h2b* expression with and without TA may appear modest in the RNA-seq data, the concordance achieved in TA+ (Figure 9) is the key validation point. *h2b* is a marker of cell proliferation. In TA−, the population advances asynchronously, introducing high biological variability that masks the true signal, rendering the measurements inconsistent. The identified RGs resolve this expected signal when TA enforces population coordination.

*wnt11a* plays a key role in the early adult development in the model cestode *Hymenolepis* (Ref 41, DOI: [10.1002/dvdy.712](https://doi.org/10.1002/dvdy.712) and DOI: [10.1242/dev.204781](https://doi.org/10.1242/dev.204781)). Its expression marks the transition zone between the neck and strobila, where segment formation begins. In this region, *wnt11* is active in muscle domains that help define segment boundaries and establish anterior–posterior polarity. As development progresses, *wnt11* continues to show polarized expression in each segment, supporting its role in organizing the body axis.

The observed expression patterns, including variability in *wnt11a* and the reliance on TA for *h2b* alignment, demonstrate that *rpl13* and *ef1α* successfully account for background noise while accurately reflecting dynamic developmental processes when the biological context is relevant.

The authors make valid claims that some of the 8 RGs could be differentially expressed in different biologically relevant contexts. They recommend rpl13 and ef1a as RGs. They perform fold change analysis using the combination of those primer pairs as normalization controls as an example of how gold-standard qPCR analysis should be performed. *I think this was an important demonstration considering how relying on a single RG can produce results that are driven by normalization. I recommend that the authors strengthen the language discussing this (paragraph at Line 395 or in the discussion). I think it would be valuable for them to explicitly state that using the geometric mean of two endogenous controls is highly advisable.*

Thank you for highlighting the importance of our demonstration and for recommending emphasis on best practices in qPCR normalization. We agree that the language surrounding this best practice should be strengthened.

While the geometric mean of three endogenous controls is often cited as the methodological gold standard, our exhaustive stability analysis demonstrated that only *rpl13* and *ef1α* maintained the necessary unconditional stability across the three developmental phases studied and culture conditions (TA+ and TA−). We mention (line 524) that although *mapk3* ranked highly in stability, we do not recommend its use because its expression appears sensitive to TA-dependent modulation. The expression of the glycolytic enzyme *pgk1*, also stable, may be influenced by the standard glucose concentration in the RPMI culture medium.

The reformulation (line 444), in the Discussion, should reinforce this key methodological point:

“Based on this demonstration, we explicitly state that using the geometric mean of two validated RGs (*rpl13* and *ef1α*) is highly advisable. While three controls are often recommended, rigorous analysis confirmed that only these two maintained the necessary unconditional stability required for reliable results in this system, as other stable candidates were excluded due to potential TA-dependent modulation or media bias. This gold-standard practice minimizes bias and improves the reliability of qPCR results by ensuring that results are not driven solely by normalization against a single, potentially unstable, reference gene.”

*I have only one concern that needs to be experimentally addressed- potential DNA contamination.* *The authors state that they DNase treat the extracted RNA but there is no data showing that these primers are not detecting DNA. In my experience, stable qPCR data can be erroneously obtained when there is DNA contamination. Furthermore, DNase from commercial kits are not always effective, especially if the treatment was done in-column. There are no details about primer design (at intron-exon boundaries or flanking large introns) to alleviate these concerns. Figure 4 showing the melting curves for a single product would not detect DNA contamination if the genomic product is the same size (i.e. primers were designed within single exons). The state of the genome may not be reliable enough for such stringent design parameters anyway. The solution is a simple one: perform end-point RT-PCR with and without reverse transcriptase (+/- RT) and show the gel. I would be perfectly satisfied with data showing this for rpl13, ef1a, h2b, and wnt11a only.*

We thank the reviewer for this critical and constructive comment regarding the possibility of DNA contamination in our qPCR assays. We conducted qPCR reactions with (+RT) and without (–RT) reverse transcriptase for *rpl13*, *ef1a*, *h2b*, and *wnt11a*. We added *gapdh1* and *mdh*, as these two and *wnt11a* are the only three in our collection of PCR targets for which primer design spans beyond one exon. Most primer pairs would not discriminate between cDNA and gDNA. **Table R1** [Primers](https://docs.google.com/spreadsheets/d/1bxUzj4ote-BpcZ4WySa3BIq3EyXMYmw8jWN-k7g4uNc/edit?usp=sharing) shows primers for all candidate RGs and both targets, information about exon location, and expected product sizes. Contrary to our expectations, amplicons were observed in the –RT reactions (**Fig. R1**), indicating the presence of trace genomic DNA (gDNA) contamination despite DNase treatment (a possibility stated by the reviewer).

**Fig. R1. Assessment of contaminating gDNA by qPCR**

Electrophoresis on 2.5% agarose gel, 100V, 60 min. qPCR products of six genes: *rpl13, ef1a, wnt11a, h2b, gapdh1, mdh;* product sizes are between parentheses*.*

NS: qPCR performed with master mix without template.

-RT: qPCR of RNA after retrotranscription with High-Capacity cDNA Reverse Transcription Kit without MultiScribe Reverse Transcriptase.

+RT: qPCR of RNA after retrotranscription with High-Capacity cDNA Reverse Transcription Kit including MultiScribe Reverse Transcriptase.

qPCR reactions were done with the PowerUp SYBR Green Master Mix (Thermofisher Scientific, Waltham, MA), 10 μL reaction in the C1000 Dx Thermal Cycler with CFX96 Real-Time System (1841000-IVD, BIO-RAD).

To quantify the level of gDNA contamination, we performed qPCR under the same conditions described in the Methods section and calculated ΔCq values between +RT and –RT reactions. We estimated the relative contribution of gDNA to the total amplification signal using the Livak and Schmittgen (2001) method (Ref. 43; DOI: [10.1006/meth.2001.1262](https://doi.org/10.1006/meth.2001.1262)); N is the number of template molecules when the reaction starts:

N_−RT_/N_+RT_ = 2^−ΔCq^

According to D´haene, Vandesompele and Hellemans (2010; DOI: [10.1016/j.ymeth.2009.12.007](https://doi.org/10.1016/j.ymeth.2009.12.007)) and Laurell et *al*. (2012; DOI: [10.1093/nar/gkr1259](https://doi.org/10.1093/nar/gkr1259)), contamination with gDNA can be tolerated or considered negligible if there is a large difference in Cq values between - RT and +RT reactions. For example, a ΔCq of 5 cycles means a 32-fold difference between cDNA and gDNA, or a contribution of 3% to the measurement of a given gene expression. In our case, as shown in **Table R2** [Cq - RT+ & RT-](https://docs.google.com/spreadsheets/d/1JRmJtBDmBKGAudaC1f1ksbcTcfrCkVVVXg_Hb9sWYaw/edit?usp=sharing), the mean Cq values in the +RT reactions are between 18.86 and 25.64, with an average Cq of 21.98, and the mean Cq values in the -RT reactions are between 29.23 and 33.07, with an average Cq of 31.88. Differences in Cq are between 6.98 and 13.69. In summary, the gDNA contribution was consistently below 1% across all genes tested.

These results confirm that, although trace gDNA contamination is detectable, its quantitative impact on qPCR-based expression data is negligible. Therefore, the integrity and reliability of the reported gene expression results remain unaffected.

**Conclusions (R#1)**

The conclusions are supported and there are no over-interpretations. The public health relevance was also discussed well.

Thank you for this supportive comment on the strength and balance of our conclusions, as well as the relevance of the findings to public health. We are pleased that the discussion was found to be appropriately framed. As mentioned above, there is a brief addition to the Discussion (lines 520-523) to highlight the *in vitro* model, which strongly recommends the addition of TA for similar studies.

**Summary and General Comments (R#1)**

Taenia solium is an important human pathogen ranked by the WHO as a neglected tropical disease though little is known about gene expression changes at development stages of these parasitic flatworms. Ever growing sequencing modalities are expanding our toolkit, but there is a pressing need to perform validations at the bench, and this study is making an important contribution to that effort. This paper validates reference genes (RGs) for use as endogenous controls in qPCR. They use quality control of various primer and amplicon stability programs in addition to qPCR validations compared to RNAseq data. They narrow down a list of acceptable RGs and use them to compare changes at three developmental stages for two markers that indicate increased proliferation and increased strobilation respectively. *With a single exception (the lack of data addressing potential DNA contamination),* the rigor shown in this paper is excellent and will provide a blueprint for other researchers to assess RGs for their species of interest. The authors also demonstrate a good example of how more reliable qPCR conclusions can be drawn using more than one RG for normalization.

Thank you for this generous and thoughtful summary. We are pleased to see that our contribution to advancing molecular tools for *Taenia solium* is recognized, particularly in terms of bench-level validation. We appreciate the acknowledgment of our methodological rigor and the potential utility of our approach as a blueprint for future studies. We are confident that gDNA contamination is not an issue. We strengthened the discussion on the importance of using multiple reference genes for normalization, as recommended.

………………….

**Reviewer #2**

**Methods (R#2)**

Regarding the methods, I believe they are well designed and described with sufficient technical detail. *However, one point that raises a question is the normalization strategy: the authors normalized the RNA-seq data using DESeq2 and then calculated CPM values with edgeR. It is not typical to use normalization results from one tool, which relies on specific assumptions, and then derive CPM values with another tool based on that prior normalization.
If this approach is indeed valid, I would suggest providing a clearer explanation of the rationale and methodological details for this step.*

Thank you for pointing out this inconsistency in our RNA-seq normalization strategy. We acknowledge that combining DESeq2 normalization with CPM calculation in edgeR was a methodological oversight, and we appreciate the reviewer’s attention to this detail. To address this invalid approach, we have revised the Methods section to clarify the workflow and ensure consistency in the use of normalization tools. Lines 133-134 now state: “Counts per million (CPM) values were calculated with edgeR (32) from RNAseq raw counts.” These revised CPM values are three new tabs in the revised Supporting Information Table S2, “input_cpm_TA+_TA-”, “input_cpm_TA+”, and “input_cpm_TA-” . **Table R3** ([CPMs](https://docs.google.com/spreadsheets/d/127oXmJadur6kX6hKMaaOcYlr6Qjvj9tb7d4YkSVDhec/edit?usp=sharing)) presents both the original and the revised values, showing negligible differences - limited to the third decimal place (thousandths) - which do not affect the analyses. Table 3 (Expression of candidate RGs), which includes a column for Average log2(CPM+1) was updated with the revised CPM values.

We still verified stability calculations, and upon re-running the GeNorm and NormFinder analyses, identified an error in the TA- results that was unrelated to the CPM values. The corrected data is in **Table R4** [genorm normfinder RNAseq](https://docs.google.com/spreadsheets/d/1DCrg8BfTceTA_6i16KADm3OvC1KwiZT80afDyY2Mv5s/edit?usp=sharing), showing both original and revised values. Although Fig. 7 was updated redoing graphs corresponding to RNASeq, none of the original conclusions is affected; gene rankings remain unchanged.

**Results (R#2)**

The results are clearly presented, and the figures are well aligned with and supportive of the findings described in the text.

Thank you for this positive comment. We appreciate the recognition of our efforts for clarity.

I believe it is important that the authors *address or discuss deeply certain inconsistencies observed*. While two strong reference genes, *rpl13* and ef1a, are identified, Figure 8A shows that for h2b in TA–, the RT-qPCR results do not replicate what was observed in the RNA-seq data. Moreover, when using rpl13 as a normalizer, a significant downregulation of h2b is detected in EV vs. PRO, whereas the RNA-seq results show the opposite trend. It would be valuable if the authors could explain or provide possible reasons for these discrepancies.

We thank the reviewer for pointing out the discrepancy in *h2b* expression between RT-qPCR and RNA-seq under TA− conditions, and asking for explanations. This observation is central to understanding the biological dynamics of our system, which deserves emphasis.

The divergence reflects differences in sampling strategy and underlying biological heterogeneity. RNA-seq libraries were prepared from pooled samples (4 parasites per replicate), which averages out individual variability. RT-qPCR measurements, in contrast, were performed on ten individual parasites, capturing inter-individual differences.

*h2b* is a histone gene tightly linked to chromatin remodeling, cell proliferation, and developmental transitions, such as in larval metamorphosis and strobilation. In TA− conditions, parasites are likely in asynchronous physiological states, leading to variable *h2b* expression across individuals. Since *h2b* expression is sensitive to the cell's physiological state and cycle, its accurate measurement requires a synchronized population.

Only under TA+ conditions, RT-qPCR and RNA-seq trends for *h2b* converge, indicating that TA acts as a synchronizing factor. It promotes or sustains development toward the adult stage. This is supported by increased evagination rates and the stability of *mdh*, a marker of adult-like metabolic activity, which is stated in the Discussion. Reviewer #1 noted that validating RGs biologically is challenging without clear benchmarks—but we argue that TA+ provides such a benchmark, as it reflects the natural *in vivo* environment of the parasite in the host intestine, where bile acids like taurocholate are present.

Hence the discrepancy in *h2b* under TA− is not a limitation of the RGs, but rather a reflection of biological heterogeneity or asynchronous development. The convergence under TA+ supports our hypothesis that TA synchronizes the population into coordinated transcriptional changes, improving both methodological reproducibility and biological relevance.

We revised the Discussion to strengthen this idea (highlighted additions):

- Line 481. Change in subtitle: **The relevance of TA for *T. solium* cyst-to-early adult development**

- Lines 511-523. "Although the reference genes *rpl13* and *ef1α* remained stable across all developmental phases and regardless of the presence of TA, their ability to validate biological results did depend on this condition. The normalization of *h2b* expression using their geometric mean only aligned with RNA-seq data under TA+ conditions. In contrast, *wnt11a* expression was consistent with RNA-seq in both TA+ and TA− (Fig. 8 and Fig. 9). This suggests that these reference genes more accurately capture biological variability under TA+ conditions, which better mimic the host environment. The concordance of *h2b* and *wnt11a* expression with RNA-seq data in TA+ cultures may reflect a more coordinated transcriptional program induced by TA as a synchronizing factor. This contrasts with the apparently asynchronous expression pattern observed in TA− cultures. Thus, while the cultures can be sustained in the absence of TA, its inclusion is essential for capturing the coordinated transcriptional dynamics associated with the adult transition and for enhancing the biological relevance of the assays.

In line with the previous comment, if possible, the *inclusion of RT-qPCR results generated from the same RNA samples used for RNA-seq would greatly strengthen the robustness and consistency of the direct comparison between both approaches*. I understand this may not be feasible if no RNA remains from the original experiments. In that case, *I would suggest acknowledging this point as a limitation of the study, and mention that including RT-qPCR data generated from the same RNA samples used for the RNA-seq experiments would have been particularly valuable.* Concerning that this approach would have provided a more direct and reliable basis for comparison, while also helping to disentangle differences that may simply reflect the inherent biological variability introduced when experiments are replicated independently.

We are sincerely grateful for this valuable and thoughtful suggestion, which aligns perfectly with best practices for comparative transcriptomics and would greatly strengthen the consistency between our RNA-seq and RT-qPCR data. We fully agree that the inclusion of RT-qPCR results generated from the exact same RNA samples used for RNA-seq (which as anticipated are no longer available) would have provided the most direct basis for technical comparison. Such comparison would have helped to precisely separate technical measurement variation from inherent biological variability. However, we propose that the implemented methodology, while not ideal for a direct technical comparison, paradoxically strengthens the biological robustness and general applicability of the reference genes (RGs) we selected, *rpl13* and *ef1α*, successfully validated across two distinct biological datasets.

Despite the biological separation, the selected RGs demonstrated consistent stability, ranking among the top four most stable transcripts in RT-qPCR across all TA+ and TA− conditions. Moreover, our RT-qPCR results obtained from an independent cyst collection align with the RNA-seq findings under TA+ conditions and further support the notion that TA acts as a synchronizing factor among individual cysts, mitigating the natural biological variability associated with batch effects.

Importantly, both techniques were designed with different objectives and preparation strategies: While the culture design is the same in both cases (the same three developmental phases, each with and without TA), in RNA-seq, to capture an averaged transcriptomic profile, samples were pooled from four separately generated cDNAs for each of three replicates per study group (phase and culture condition). On the other hand, RT-qPCR relied on ten individual measurements per study group from independent cultures to account for the biological heterogeneity that may exist among individual cysts by treating each cyst as the minimal biological unit for analysis. These different approaches in sampling allow a better representation of the inherent biological variability, which validates the robustness of the proposed RGs.

**Conclusions (R#2)**

The conclusions are appropriately formulated and consistent with the results obtained.

We appreciate the recognition of our efforts for clarity.

**Summary and General Comments (R#2)**

I found this work to be both interesting and relevant, particularly in regard to the questions it addresses. The search for suitable reference genes as normalizers for RT-qPCR experiments is highly important, as it directly impacts the accuracy and reliability of gene expression analyses. *In this context, it would also be valuable for the authors to discuss certain methodological choices and potential limitations, such as the normalization strategy used for RNA-seq and the possibility of including RT-qPCR data from the same RNA samples.* Addressing these points would further strengthen the study and provide a clearer basis for comparison between RNA-seq and RT-qPCR results.

Thank you for this supportive assessment. We appreciate the recognition of the relevance and impact of our study. We acknowledge the importance of clarifying our RNA-seq normalization strategy and have made the necessary corrections to the Methods section accordingly (lines 133-134).

We have now addressed this point in the revised manuscript by discussing the inherent trade-off between using the same RNA samples for both RNA-seq and RT-qPCR (thereby strengthening direct comparability) and ensuring the robustness of the selected reference genes by validating them through independent technical approaches and across biologically distinct samples, which unavoidably introduces some variability.
