# Supplementary material for: Validation of reference genes for RT-qPCR relative expression analysis during cyst-to-early adult development of Taenia solium
Source: PLoS Negl Trop Dis. 2026 Jan 2;20(1):e0013893. doi: 10.1371/journal.pntd.0013893 (PMC12900441; doi:10.1371/journal.pntd.0013893)
Supplement: Attachment — Submitted filename: Response to Reviewers R2.docx [file pntd.0013893.s008.docx]

**PNTD-D-25-01312R1
Validation of reference genes for RT-qPCR relative expression analysis during cyst-to-early adult**

Dear editors,

Please find below the response to the comment by reviewer #1. We thank the reviewer for helping us to comply with reporting guidelines while adding interest for readers in our results.

C. Guerra G (for the authors).

…………………...

**Summary and General Comments**

**Reviewer #1**

**Thank you for addressing all my concerns. The way the data is presented suggests that the authors do not intend to include Table R1, Table R2 and Fig. R1 in the manuscript and that they were meant for reviewers only. Please include them in the supplement. Table R1 can be merged into Table S1 easily. While I agree that the genomic contamination did not significantly alter the findings in this paper, readers may be surprised to discover that Cq values between 29 and 33 can be obtained in the ABSENCE of cDNA. Cq values tend to be lower for RGs than for any given gene of interest. Thus, I believe it is valuable to report this.**

Thanks for the suggestion to add the two tables and the figure as Supporting Information Files; we fully agree that it is better to mention the gDNA contamination and its lack of impact on the results. Table R1 (primer location) is now a tab in Supplementary Information S1, Fig R1 (gel electrophoresis) is S2, Table R2 (gDNA contribution to signal) is S3, and the former S2 and S3 files are now S4 and S5. The five legends for the Supplementary Information files are after the References, following editorial guidelines.

The revised manuscript includes these additional lines to direct readers to the added supplementary material:

Line 163-164 in Methods:

“For most genes, primers were located within single exons (S1).”

Lines 266-274 in Results:

“To evaluate potential gDNA contamination, RT‑qPCR reactions with and without reverse transcriptase (+RT/–RT) of representative genes were performed (S2). Amplicons detectable in –RT controls confirmed the presence of trace gDNA despite DNase treatment. However, mean Cq values in –RT reactions (29–33) were consistently 7–14 cycles higher than in +RT reactions (19–26) (S3), corresponding to <1% contribution to total signal when calculated using the comparative ΔCq method [(42)](https://www.zotero.org/google-docs/?xJvabJ). According to established criteria [(43,44)](https://www.zotero.org/google-docs/?SQKVI9), such differences indicate negligible contamination.”

Lines 558-561 in Discussion:

“MIQE 2.0 (61) guidelines call for disclosure of primer location and validation data (S1, S2). Although most primer pairs in our study were located within single exons, gDNA contamination had no meaningful impact on the accuracy of our strategy or the reliability of the expression data.”
